# Ultra-deep sequencing of VHSV isolates contributes to understanding the role of viral quasispecies

**DOI:** 10.1186/s13567-015-0298-5

**Published:** 2016-01-08

**Authors:** Anna A. Schönherz, Niels Lorenzen, Bernt Guldbrandtsen, Bart Buitenhuis, Katja Einer-Jensen

**Affiliations:** Department of Molecular Biology and Genetics, Center for Quantitative Genetics and Genomics, Aarhus University, Blichers Allé 20, P.O. Box 50, 8830 Tjele, Denmark; Department of Animal Science, Aarhus University, Blichers Allé 20, P.O. Box 50, 8830 Tjele, Denmark; QIAGEN AAR, 8000 Århus, Denmark

## Abstract

The high mutation rate of RNA viruses enables the generation of a genetically diverse viral population, termed a quasispecies, within a single infected host. This high in-host genetic diversity enables an RNA virus to adapt to a diverse array of selective pressures such as host immune response and switching between host species. The negative-sense, single-stranded RNA virus, viral haemorrhagic septicaemia virus (VHSV), was originally considered an epidemic virus of cultured rainbow trout in Europe, but was later proved to be endemic among a range of marine fish species in the Northern hemisphere. To better understand the nature of a virus quasispecies related to the evolutionary potential of VHSV, a deep-sequencing protocol specific to VHSV was established and applied to 4 VHSV isolates, 2 originating from rainbow trout and 2 from Atlantic herring. Each isolate was subjected to Illumina paired end shotgun sequencing after PCR amplification and the 11.1 kb genome was successfully sequenced with an average coverage of 0.5–1.9 × 10^6^ sequenced copies. Differences in single nucleotide polymorphism (SNP) frequency were detected both within and between isolates, possibly related to their stage of adaptation to host species and host immune reactions. The N, M, P and Nv genes appeared nearly fixed, while genetic variation in the G and L genes demonstrated presence of diverse genetic populations particularly in two isolates. The results demonstrate that deep sequencing and analysis methodologies can be useful for future in vivo host adaption studies of VHSV.

## Introduction

Viral haemorrhagic septicaemia virus (VHSV) is an RNA virus endemic to marine and freshwater fish species. It represents one of the most important viral pathogens in salmonid fish in continental Europe where it heavily affects cultured rainbow trout, causing a severe systemic disease with mortality rates as high as 90% [1] and thus resulting in extensive economical loses to the aquaculture industry [2, 3].

VHSV is a single-stranded RNA virus of negative polarity that belongs to the genus *Novirhabdovirus*, within the family *Rhabdoviridae*. Genetic analyses show that the virus clusters into four major phylogenetic clades classified as genotypes I–IV with further subdivision of genotypes I and IV. Genotypes are correlated to geographic regions rather than host species with genotype I–III circulating in Europe and genotype IV circulating in North America and Asia [4–7]. Although these phylogenetic analyses cannot define host species specificity, they do demonstrate that isolates of VHSV from rainbow trout are principally members of genotypes Ia, Ic and Id, and the isolates adapted to marine host species are members of genotypes Ib, Ie and II–IV. The genetic differentiation of rainbow trout and marine adapted isolates is also phenotypically manifested in different pathogenicity patterns towards the two host groups [8]. Rainbow trout adapted isolates are highly pathogenic to rainbow trout but show very low or no pathogenicity in marine fish species and vice versa.

Moreover, it has been revealed that rainbow trout adapted isolates have evolved from the marine environment and are the result of cross-species transmission followed by subsequent host adaptation [5, 9, 10]. Cross-species transmission from marine hosts to cultured rainbow trout has occurred several times. Recently, cross-species transmission events have been reported in rainbow trout cultured in marine waters of Finland [11, 12], Norway [13] and Sweden [14, 15].

The repeated emergence of VHSV into cultured rainbow trout illustrates the high evolutionary potential of the virus. This is a feature common to all RNA viruses [16, 17]. RNA viruses have high replication rates, large population sizes, and exceptionally high mutation rates commonly ranging from 10^−3^ to 10^−5^ mutations per nucleotide, per replication [16–18]. The high mutation rates result from an error-prone RNA polymerase lacking proofreading abilities [18, 19]. As a consequence, RNA virus populations sustain high genetic diversity, most likely resulting in the formation of a quasispecies. A quasispecies is composed of a dominant nucleotide sequence and a spectrum of low frequency variants arising from mutations during virus replication [18]. While appearing a wasteful strategy, this feature enables an RNA virus to rapidly adapt to environmental changes or new hosts. However, very little is known about viral population structures and dynamics, especially the low-frequency mutant spectrum.

Until recently, viral evolutionary dynamics were investigated based on consensus genome sequencing, ignoring that viral samples actually represent complex, heterogeneous populations, of non-identical genome sequences. Consensus sequencing, however, only identifies the dominant or major viral sequence present in a sample but is uninformative about the mutant spectrum of minority variants present in the population [20, 21]. Consequently, consensus genome sequences provide an incomplete picture of the within- and between-host viral evolutionary diversity during cross-species transmission and adaptation.

In contrast Next-Generation Sequencing (NGS) techniques provide a rapid and cost-effective analysis of viral population diversity at an unprecedented level of detail [20, 22–24]. The great depth of resolution and high-throughput nature of NGS platforms allows investigation of viral evolution at the within- and between-host scale.

The main focus of this study was to develop a VHSV specific NGS protocol applicable across viral genotypes with the aim of understanding the evolutionary potential of VHSV through characterization of its viral quasispecies. An overlapping long range PCR amplification protocol specific to VHSV was developed targeting isolates of genotype I–III. The nucleotide sequences of four cell culture passaged VHSV isolates were analysed using the Illumina HiSeq sequencing platform and coverage rates between 0.5 and 1.9 × 10^6^ were achieved allowing characterization of the viral population diversity in all four isolates of VHSV.

## Materials and methods

### Virus isolates and viral propagation

Deep sequencing of viral populations was conducted using four VHSV isolates (Table 1): two isolates originating from freshwater cultured rainbow trout (DK-3592b, DK-9895174), and two isolates originating from Atlantic herring (4p168, 1p49). Both rainbow trout isolates belong to genotype I whereas the two marine isolates belong to genotypes II (1p49) and III (4p168). All four viral stocks were propagated in bluegill fry cells (BF-2; [25]) as described earlier [26]. When complete cytopathic effect (CPE) was observed, cell medium was harvested, filtered through a 0.2-µm Minisart filter (Bie & Bernsten) and the filtrate was centrifuged at low speed (5000 RPM) for 30 min at 4 °C to remove cell debris. Subsequently, the supernatant was recovered and subjected to ultracentrifugation at 86 000×*g* for 2 h at 4 °C to pellet viral particles. The pellet was harvested and stored at −80 °C or directly subjected to RNA extraction.

**Table 1:** Data related to the four viral haemorrhagic septicaemia virus isolates used in this study.

Isolate	Genotype	Host	Geographic origin	Year of isolation	Original sample type	References	Cell culture passage
DK-3592b	Ia	Rainbow trout	Denmark	1985	Tissue pool, fish pool	[28, 39, 40]	8 Pass BF2
DK-9895174	Ia	Rainbow trout	Denmark	1998	Tissue pool, fish pool	[5]	5 Pass BF2
1p49	II	Atlantic herring	Baltic Sea	1996	Tissue pool, fish pool	[8]	1 Pass EPC, 5 pass BF2
4p168	III	Atlantic herring	Skagerrak	1997	Tissue pool, fish pool	[41]	8 Pass BF2

### RNA extraction

Total RNA was extracted from replicate samples for each isolate using the RNeasy Mini kit (Qiagen) following manufacturer’s recommendations for extraction of RNA from cell culture. Total RNA from each replicate was eluted in 30 µL nuclease-free water that was treated with DEPC (Qiagen) and finally pooled. Two microliters were used to quantify the concentration of RNA; the remainder was stored at −80 °C. The concentration of extracted RNA was determined using a spectrophotometer (NanoDrop, Thermo Scientific) and the final concentration of the pooled samples was in range of 16–40 ng/µL per isolate.

### Reverse transcription

Reverse transcription (RT) of the full-length VHSV genome was performed using the SuperScript III First-Strand Synthesis System for RT-PCR (Invitrogen) and a VHSV genome specific primer (Table 2). RT was performed following manufacturer’s recommendations. Briefly, 1 µL cDNA primer (0.01 mM) and 1 µL dNTPs (10 mM) were added to 8 µL total RNA, incubated at 65 °C for 5 min and placed on ice. Subsequently, 10 µL cDNA synthesis mix (2 µL 10× buffer, 4 µL MgCl_2_, 2 µL DTT, 1 µL RNase OUT, 1 µL SuperScript III reverse transcriptase) were added and incubated at 50 °C for 50 min, 85 °C for 5 min, and placed on ice. Finally, 1 µL RNase H was added followed by incubation at 37 °C for 20 min to remove the original viral RNA from the new synthesized cDNA. In total, 20 µL of full VHSV genome length cDNA was synthesized and either stored at −80 °C or immediately subjected to PCR amplification.

**Table 2:** Primers used for RT-PCR amplification.

Name	Sequence	Amplicon (length in bp)	Overlapping region
VHSV_Frag1I_nt18_+s*	GAGTTATGTTACA**R**GGGACAGG		
VHSV_Frag1_nt2815_−s	CGATTGTAG**Y**AGTCCTTCGC	Amplicon 1 (2797 bp)	Amplicon 1–2 = 274 bp
VHSV_Frag2_nt2541_+s	GAGAAGATTGACTTCGGGAC		
VHSV_Frag2_nt6250_−s	CGTAGGTAGGAACCCTGTC	Amplicon 2 (3709 bp)	Amplicon 2–3 = 790 bp
VHSV_Frag3_nt5460_+s	TACTGGAACTTGGCCTCACA		
VHSV_Frag3_nt8287_−s	TGTGTCCGCCAAATGGTGTA	Amplicon 3 (2827 bp)	Amplicon 3–4 = 431 bp
VHSV_Frag4_nt7856_+s	GATGATTGTCTCCACCATGAA		
VHSV_Frag4I_nt11032_−s	TCTCCAAATGGAAAGAAGGACT	Amplicon 4 (3176 bp)	

Names include +s or −s, which reflects the positive or negative sense orientation, respectively.

* Primer used for reverse transcription of the genomes.

### Polymerase chain reaction (PCR) and DNA purification

**Figure 1:**
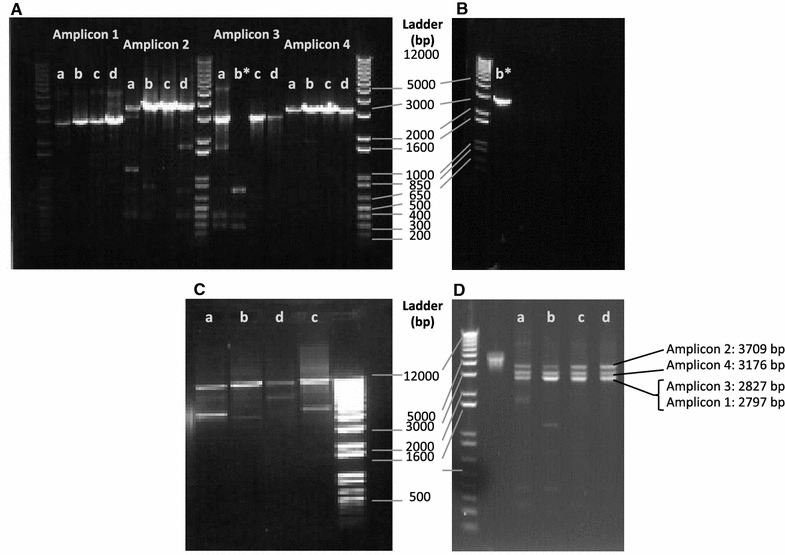
**Generation of the four overlapping amplicons (1–4) to cover the entire viral genomes of each isolate.** The VHSV isolates, are here abbreviated *a* DK-3592b; *b* DK-9895174; *c* 1p49; *d* 4p168. The individual RT-PCR reactions are shown in **A**; **B** re-amplified Amplicon 3 of sample b*; **C** amplification of the whole genome RT-PCR reaction; **D** pooled amplicon samples (1:1:1:1) for each of the VHSV samples used for library preparation.

PCR amplification of the full length VHSV genome was performed using the Platinum^®^ Taq DNA Polymerase High Fidelity kit (Invitrogen) and single primer set amplifying a 11,014 bp region covering all 6 open reading frames, all intergenic regions and partial regions of the leader and trailer sequence (sense primer VHSV_Frag1I_nt18_+s: 5′-GAG TTA TGT TAC ARG GGA CAG G-3′; antisense primer VHSV_Frag4I_nt11032_-s: 3′-TCT CCA AAT GGA AAG AAG GAC T-5′). Amplification was performed for all four isolates but full-length genome amplification could only be established for 3 of the isolates (DK-3592b, DK-9895174, 1p49) and with unwanted smaller fragments (Figure 1). To maximize coverage depth, the genome was divided into four amplicons that were numbered sequentially as amplicon 1–4 starting from the 5′ end of the genome with amplicons ranging from 2797 to 3709 bp in length and overlapping with the adjacent amplicons by 274–790 bp (Table 2). Primers were designed to target conserved regions of the VHSV genome irrespective of host origin and genotype (Table 2). PCR amplification was performed for each fragment and isolate separately using the Platinum^®^ Taq DNA Polymerase High Fidelity kit (Invitrogen) and the corresponding primer sets. Amplification was conducted in a total volume of 50 µL in a MX Pro-Mx3005P thermocycler. Reactions contained 2 µL cDNA, 5 µL 10x high Fidelity PCR buffer, 1 µL dNTPs (10 mM), 2 µL MgSO_4_ (50 mM), 0.2 µL Platinum^4^ Taq High Fidelity Polymerase, 1 µL sense primer (0,01 mM), 1 µL antisense primer (0.01 mM), and 37 µL nuclease free water. Amplicons were produced using the following cycling program: 94 °C for 1 min, followed by 25 cycles of 94 °C for 30 s, 58 °C for 30 s, and 68 °C for 4 min, with a final step of 68 °C for 5 min. Individual PCR products were visualized using agarose gel electrophoresis running 6 µL on a 1% agarose gel. A total of 30 µL of the remaining amplified DNA was purified using the QIAquick PCR Purification kit (Qiagen) following manufacturer’s recommendations. Purified DNA was eluted in 50 µL EB buffer (10 mM Tris·Cl, pH 8.5). DNA concentration was determined by fluorescence detection using a Qubit^®^ Fluorometer (Invitrogen) and the Quant-iT™ dsDNA BR Assay kit (Life Technologies). Subsequently, amplicons of the same isolate were adjusted to equivalent concentrations, combined to one sample and stored at −20 °C.

### Library preparation and sequencing

The paired end library preparation, as well as Illumina HiSeq (2 × 100) sequencing of the four samples, was performed on contract basis by Beckman Coulter Genomics. Briefly, library construction was performed using sheared DNA as input to the SPRI-TE system and LC cartridges (Beckman Coulter, Inc.). TruSeq PE indexes (Illumina) were added by ligation, the libraries were amplified by PCR, and purified with AMPure XP beads (Beckman Coulter, Inc.). The libraries were clustered on Illumina High-Output v3 Flowcells and sequenced using a HiSeq model 2500 sequencer (Illumina). Beckman Coulter Genomics performed the primary software analysis using the Illumina instrument application Real-Time Analysis software, and hereby converted raw images into base calls. Demultiplexing was then performed resulting in FASTQ-formatted read-groups using the CASAVA software package.

### Bioinformatics analysis

The demultiplexed FASTQ files provided by Beckman Coulter Genomics were analysed in a single workflow using the specified tools (subsequently marked with “”) which all are available in the CLC Genomics Workbench V7.5.1. The paired reads were trimmed using the “Trim sequence” tool 4 times for removal general adapters, PCR adaptors, PCR primers, and finally ambiguous nucleotides (maximum number of ambiguities = 2) as well as nucleotides with low quality scores (limit = 0.05). The virus isolates DK-3592b and DK-9895174 were mapped against a reference sequence of DK-3592b (NCBI Accession number KC778774), while the isolates 1p49 and 4p168 obtained from herring were mapped against a VHSV isolate from sea-reared rainbow trout (strain FA281107; NCBI Accession number No EU481506). The following default parameters were used during mapping of reads to their reference sequence: mismatch cost = 2; insertion cost = 3; deletion cost = 3; length fraction = 0.5; similarity fraction = 0.8. The mapped reads were subsequently realigned and explored using the “InDel and Structural Variation Detection” tool. The hereby-generated guidance variant track was subsequently used during an additional realignment of the reads. Consensus sequence and alignment of these against their respective reference sequences were performed using the “Extract Consensus sequence” and “Create Alignment” tools, respectively.

Mapping efficiency and coverage of the individual samples were determined using the tools “Create Detailed Mapping Report” and “Create Statistics for Target Regions”, respectively.

Finally, single- and multiple nucleotide variants and short insertions and deletions were called using the “Low Frequency Variant Detector” tool where broken pairs and non-specific read matches were ignored. The minimum coverage, count and frequency were set to 1000, 200 and 1%, respectively, while the required significance was 0.01% and the relative read direction filter significance was set to 10^−19^.

To reduce the number of false positives due to PCR errors, quality filtering was performed using the following conservative thresholds: equality in forward and reverse reads at least 0.35, average quality at least 30, and statistical testing of read position and read direction probability should exceed 10^−12^. Since marginal variants could still be related to cell culture passaging, a final frequency-filtering criterion was applied including polymorphic sites with intermediate allele frequency (5–95%) but excluding polymorphic sites with minor allel frequency below 5%. For each of these filtering steps, the functional consequence was determined by translating the encoded CDS’s using the “Amino Acid Changes” tool.

### Statistical analysis

Patterns of genetic variation within and between isolates were investigated using the statistical program R (version 3.1.0 R Core Team, 2014). All analyses conducted were based on variant calls that passed quality control criteria. Only polymorphisms with allele frequencies between 5 and 95% were retained for analysis. The frequency filter was applied to ensure that variants investigated represented polymorphisms of the original isolate instead of variants introduced during cell culture, RT-PCR amplification or sequencing and to ensure that those variants represented genetic variation present within the original viral population.

To identify whether genetic variation differed between genes or isolates, a generalized linear model comparison analysis was conducted assuming that the number of substitutions, including both synonymous- and non-synonymous substitutions, as the response variable followed a Poisson distribution. The explanatory variables were gene ($g_{i}$: 6 levels representing genes encoding the nucleoprotein (N), phosphoprotein (P), matrix protein (M), glycoprotein (G), non-structural protein (NV), and the RNA-dependent RNA polymerase (L)), and isolate ($i_{j}$: 4 levels representing the isolates DK-3592b, DK-9895174, 1p49, 4p168), as well as a regression coefficient taking into account the effect of the length of the gene in nucleotides. The full linear model was:

$$\log \left( {\lambda_{ij} } \right) = g_{i} + s_{j} + log(l_{i} )$$

where $\lambda_{ij}$ is the parameter of the Poisson distribution, *g*_*i*_ is the effect of gene *i*, *s*_*j*_ is the effect of isolate *j*, log (*l*_*i*_) is the logarithm of the length of gene *i*. The coefficient of the logarithm of the gene length was fixed to 1. Three models were investigated: (1) the full model including all explanatory variables; (2) a reduced model without gene as explanatory variable; and (3) a reduced model without isolate as explanatory variable. The generalized linear models were fitted using the “*glm*” function from the R package “*stats*”. Models were compared using the “*anova*” function in R.

To identify contrasts of genetic variation between genes, genes were divided into two groups: (1) genes with low genetic variation (N, P, M, NV); (2) genes with high genetic variation (G, L). The generalized linear model comparison analysis was repeated, but with gene grouped into 2 levels (high genetic variation, low genetic variation), isolate (4 levels) and gene length included as explanatory variables, investigating whether genetic variation in the G and L gene is higher compared to the remaining genes. A Bonferroni correction was applied to account for all possible groupings of 6 genes. The total number of possible groupings is given by the sixth Bell number minus 2, B_6_ = 203, that is correcting for 201 simultaneous (implicit) tests. The two cases that are subtracted are the case of each gene having a separate effect and the case of all genes having the same effect. When looking at contrasts among genes, this is the most conservative choice.

To identify contrasts of genetic variation between isolates, isolates were divided into two groups: (1) isolates with low genetic variation (DK-3592b, DK-9895174, 4p168); (2) isolates with high genetic variation (1p49). The generalized linear model comparison analysis was repeated, but with isolate grouped into 2 levels (high genetic variation, low genetic variation), gene (6 levels) and gene length included as explanatory variables, investigating whether genetic variation in 1p49 is higher compared to the reaming isolates. The Bonferroni correction was adjusted to correct for all possible groupings of 4 isolates (B_4_ = 15).

## Results

### Amplicon amplification

**Figure 2:**
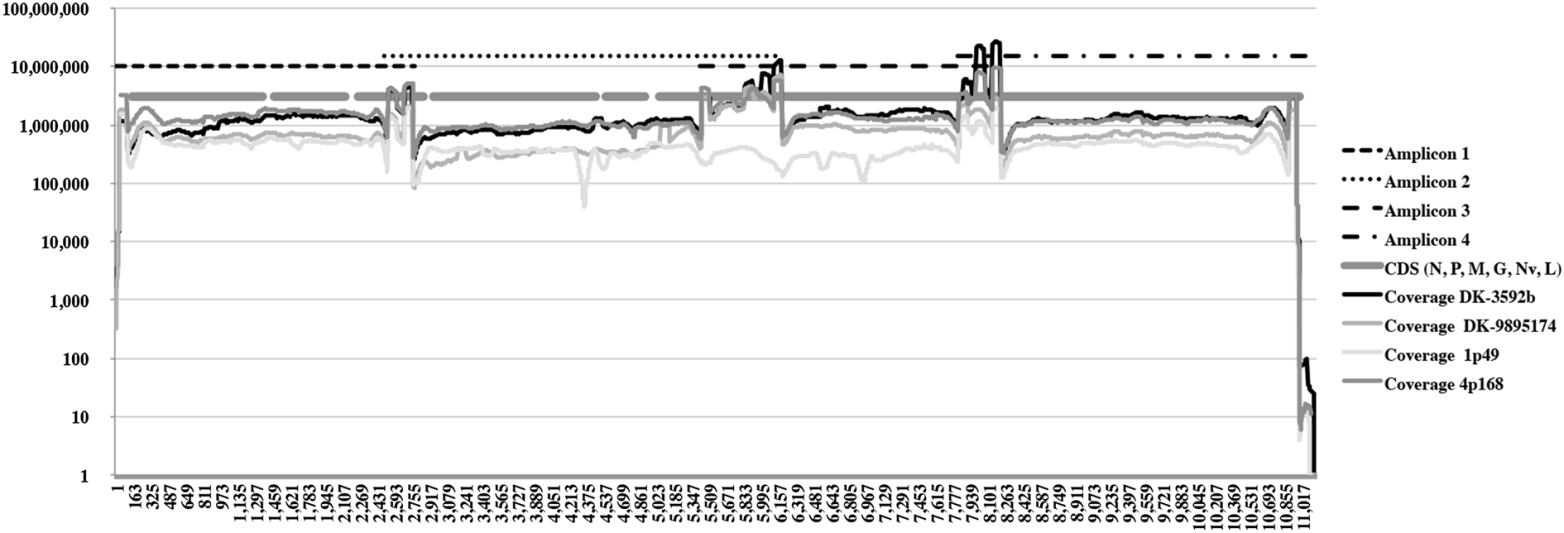
**Coverage of mapped reads across the whole VHSV genomes for all four isolates.** The four overlapping amplicons (1–4) and CDS regions are visualized according to the genome numbers of Acc No KC778774. The obtained mapping coverage for each nucleotide position of the individual virus isolates is shown using a logarithmic scale.

Based on agarose gel electrophoresis, amplicons of expected size were synthesized by RT-PCR. However, small amounts of shorter amplicons were also observed (Figure 1A). Attempts to extract the DNA bands with the expected amplicon size resulted in too low a concentration of purified DNA. Instead, a 1:1:1:1 amplicon mixture was made from purified, but not gel-extracted samples, and therefore included some amplicons that were shorter than desired (Figure 1D). The obtained coverage data (Figure 2) showed unexpected increase in coverage (up to 20×) at the first nucleotide upstream of primer 3′ end and at the sequences in the overlapping regions of amplicon 1 and 2, amplicon 2 and 3, and amplicon 3 and 4, respectively. This finding was unexpected since the performed PCR amplifications were performed in individual tubes, and not as multiplex reactions. Whether a minor cross contamination of primer pairs may have occurred either during synthesis at the producer or during handling of the primers in the lab remains to be determined.

### Coverage and mapping efficacy

The number of generated raw paired-end reads for DK-3592b, DK-9895174, 1p49 and 4p168 was 266.4 × 10^6^, 127.6 × 10^6^, 76.4 × 10^6^ and 224.4 × 10^6^, respectively. Trimming removed between 3.2 and 6% of the reads resulting in 260.6 × 10^6^, 120.2 × 10^6^, 71.8 × 10^6^ and 212.9 × 10^6^ reads, respectively.

Based on a BLAST search two different reference genomes were identified. Isolate DK-3592b (genotype I) was identified as an appropriate reference genome for isolates DK-3592b and DK-9895174 (both genotype I), whereas isolate FA281107 (genotype III) was identified as the best reference genome for isolates 1p49 (genotype II) and 4p168 (genotype III).

Mapping efficacy of isolate DK-3592b, DK-9895174, 4p168 to their respective reference genomes was above 97%) and contained comparable fractions of paired reads (above 88%). The fourth isolate (1p49) was approximately 10% lower with respect to the number of generated reads as well as the mapping efficiency (Table 3).

**Table 3:** Read mapping of trimmed reads against a reference genome from a VHSV isolate of same host adaption environment.

Reference genome	DK-3592b (Acc No KC778774)				FA281107 (Acc No EU481506)			
Virus isolate	DK-3592b		DK-9895174		1p49		4p168	
	No of reads	In %	No of reads	In %	No of reads	In %	No of reads	In %
Mapped reads	257 431 536	98.8	117 684 334	97.8	63 880 245	89.0	209 126 393	98.2
Not mapped reads	3 179 450	1.2	2 611 384	2.2	7 936 247	11.0	3 799 011	1.8
Reads in pairs	236 329 444	90.7	106 731 672	88.7	56 208 078	78.2	197 135 466	92.6
Broken paired reads	21 102 092	8.1	10 952 662	9.1	7 672 167	10.7	11 990 927	5.6
Total reads	260 610 986	100.0	120 295 718	100.0	71 816 492	100.0	212 925 404	100.0
Average coverage	1 871 006		959 252		528 148		1 729 031	

The average coverage was very high for all four isolates but with some variations. The average coverage depths of isolate 1p49 (5.28 × 10^5^) was approximately 72% that of the isolate with the highest coverage (isolate DK-3592b with 1.9 × 10^6^ coverage). However, the coverage across the genomes appears more even for 1p49 than for the other 3 samples due to less PCR contamination/artefacts as discussed above (Figure 2).

Alignment of mapped consensus sequences to their respective reference genome revealed 99.93, 99.03 and 98.34% sequence identity for isolates DK-3592b, 4p168, and DK-9895174, respectively, but only 90.09% sequence identity for isolate 1p49.

### Variant detection

In the analysis performed, we mainly focus on polymorphisms with frequencies between 5 and 95% (Table 4), which likely reflect polymorphisms established in vivo (before cell culture propagation). Variants at population frequencies below 1% were assumed to represent polymorphisms established in vivo, variants that were established during cell culture, as well as artefacts of RT-PCR amplification and NGS sequencing [27]. The experimental design could not provide a method to distinguish between the different sources of observed polymorphisms; therefore, a relatively conservative frequency filter was applied, excluding variants below a 5% frequency threshold.

**Table 4:** Variant detection in coding and none coding regions of the four analyzed VHSV genomes.

Reference genome	DK-3592b (Acc No KC778774)												FA281107 (Acc No EU481506)											
Isolate	DK-3592b						DK-9895174						1p49						4p168					
Filtering	Nt	Aa	Nt, Q	Aa, Q	Nt, QF	Aa, QF	Nt	Aa	Nt, Q	Aa, Q	Nt, QF	Aa, QF	Nt	Aa	Nt, Q	Aa, Q	Nt, QF	Aa, QF	Nt	Aa	Nt, Q	Aa, Q	Nt, QF	Aa, QF
Leader	4	–	0	–	0	–	7	–	0	–	0	–	12	–	0	–	0	–	0	–	0	–	0	–
N	7	1	5	0	2	0	15	3	12	1	0	0	200	33	94	15	0	0	14	8	10	7	0	0
Inter N-P	3	–	1	–	0	–	3	–	2	–	0	–	11	–	6	–	0	–	0	–	0	–	0	–
P	2	1	3	1	0	0	17	4	17	3	0	0	72	11	48	7	0	0	5	2	5	2	0	0
Inter P-M	2	–	0	–	0	–	3	–	1	–	0	–	26	–	8	–	0	–	6	–	5	–	0	–
M	2	1	0	0	0	0	13	5	6	2	0	0	88	18	29	5	0	0	9	3	6	1	0	0
Inter M-G	0	–	0	–	0	–	1	–	1	–	0	–	20	–	11	–	0	–	5	–	4	–	0	–
G	22	9	17	4	5	1	60	27	48	22	9	4	286	45	161	16	7	1	32	7	15	3	2	1
Inter G-Nv	6	–	0	–	0	–	5	–	3	–	0	–	17	–	13	–	0	–	0	–	5	–	0	–
Nv	20	9	12	1	0	0	23	12	22	11	0	0	65	33	37	18	2	0	11	4	5	3	1	0
Inter Nv-L	4	–	0	–	0	–	6	–	5	–	0	–	11	–	8	–	0	–	2	–	1	–	0	–
L	15	1	8	0	3	0	85	10	65	5	0	0	869	73	464	32	91	4	51	12	38	5	0	0
Trailer^a^	0	–	0	–	0	–	0	–	0	–	0	–	0	–	0	–	0	–	0	–	0	–	0	–
Total	87	22	46	6	10	1	238	61	182	44	9	4	1677	213	879	93	100	5	135	36	94	21	3	1

The table summarizes the distribution of nucleotide (Nt) and amino acid (Aa) variants before and after filtering procedures. Two filtering procedures were applied based on read quality (Q) and variant frequency at polymorphic sites (F). Read quality filtering was applied using the following parameters: equality in forward and reverse reads should be at least 0.35, average quality at least 30, while statistical testing of read position- as well as read direction probability should exceed 10^−12^. Filtering on variant frequency was conducted to distinguish true variants from errors introduced during sequence preparation and sequencing. Only polymorphic sites with variants with intermediate allel frequencies (5–95%) were incluede whereas polymorphic sites with minor allele frequencies below 5% were excluded from analysis.

^a^Coverage of trailer was below 80×.

**Figure 3:**
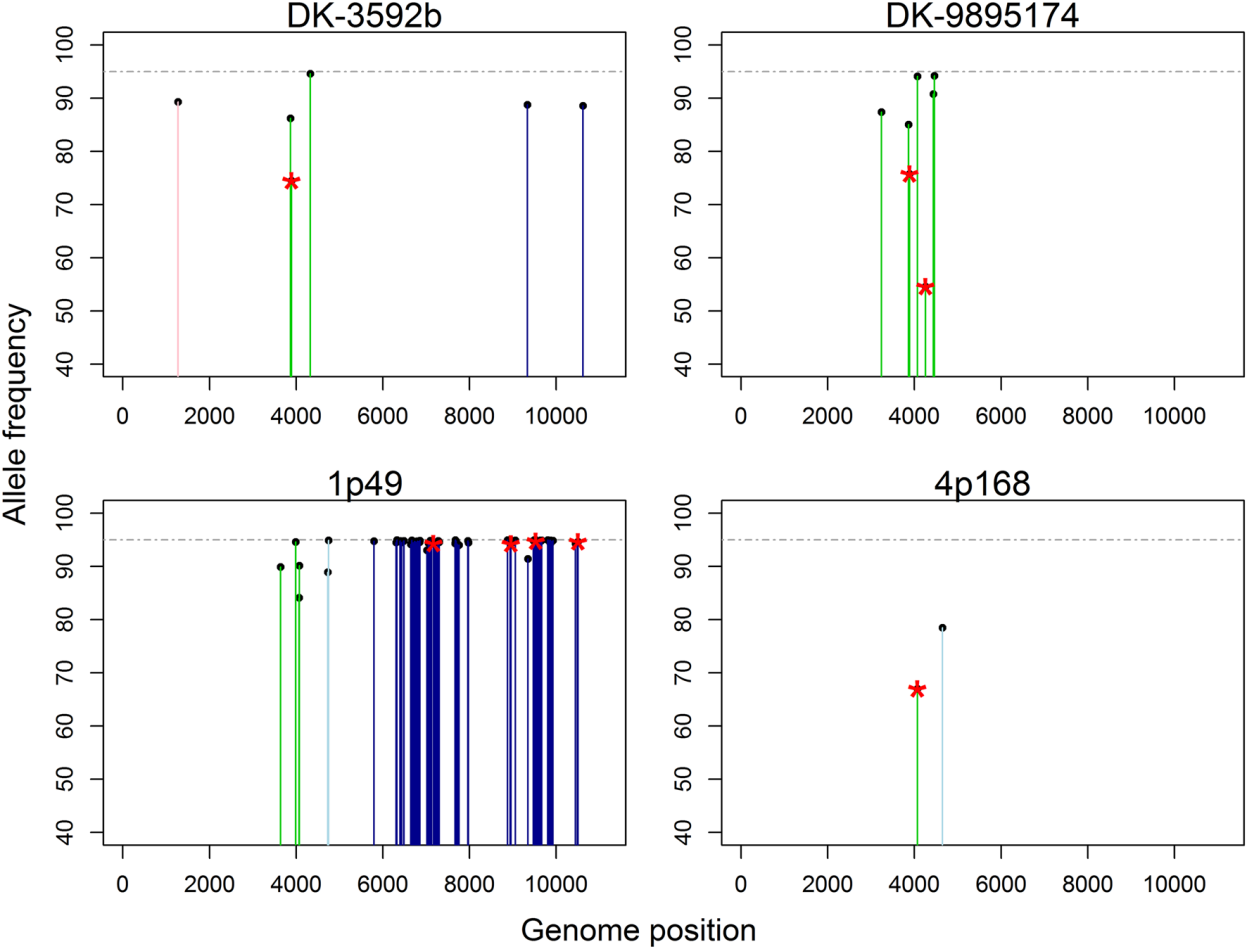
**Detected polymorphic positions with intermediate allele frequency (5–95%) representing the allele frequency of the dominant allele (frequency between 50 and 95%).** *Colours* of* vertical lines* indicate different genes:* pink* N,* yellow* P,* red* M,* green* G,* light blue* NV,* blue* L,* dotted vertical lines* intergenic regions.* Horizontal line* represents the 95% frequency threshold.* Red stars* at top of* vertical lines* indicate amino acid change in the dominant allele.

Following quality- and frequency filtering, the highest genetic variation at intermediate allele frequencies was detected for isolate 1p49 with 100 variants, followed by DK-3592b with 10 variants, DK-9895174 with 9 variants, and 4p168 with 3 variants (Table 4). Variants recorded included the minor and the major allele detected for each polymorphic side that passed quality control requirements. Thus, called variants do not represent the number of polymorphic sites detected, but rather the total allelic variation. A total of 89, 8, 7 and 2 polymorphic sites with intermediate allele frequencies were detected for 1p49, DK-9895174, DK-3592b and 4p168, respectively. Polymorphic sites were located in the N gene (DK-3592b), the G gene (all isolates), the NV gene (1p49), and the L gene (DK-3592b, 1p49). No genetic variation at intermediate allele frequencies was detected in the M and P gene, or in the intergenic regions. In Figure 3, positions of polymorphic sites across the genome are shown representing the allele frequency of the dominant variant (50–95% allele frequency). The frequency of dominant alleles was shown as some variants of minor allele frequency did not pass the quality requirements and thus were not recorded.

**Figure 4:**
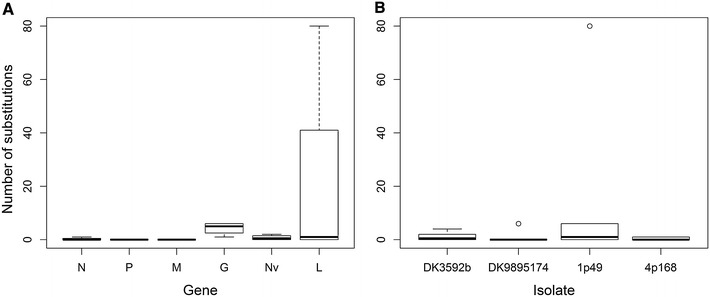
**Box plot of number of detected substitutions with intermediate allele frequencies (5–95%). Number of substitutions is represented as a count of substitution events recorded for polymorphic sites with intermediate allele frequencies (5–95%).** Accordingly, substitution events resulting into alleles with frequencies below 5% were ignored. **A** The substitution counts for the individual gene across all four isolates for each of the six genes (four counts per gene). **B** Substitution counts for the individual isolate across all six genes for each of the four isolates (six counts per isolate).

Based on Table 4 and Figure 3, the G and L genes show the highest level of genetic variation. Furthermore, isolate 1p49 shows higher genetic variation than the other three isolates. Patterns of genetic variation within and between isolates were confirmed by generalized linear model comparison analysis. Comparing the full model with the two model reductions revealed a significant difference between the full- and reduced models without gene as explanatory variable (*p* value = 9.955e^−10^) and between the full- and reduced models without isolate as explanatory variable (*p* value = 2.2e^−16^). Hence, both the genes as well as the isolates have an effect on the number of substitutions observed and the local substitution rate because gene length was included in each model as a regression coefficient. Comparison between genes of high genetic variation to genes with low genetic variation revealed that the average number of substitutions in the G and L gene was significantly higher compared to other genes (*p* value = 0.002) (Figure 4). In addition, comparison between isolates of high genetic variation with isolates of low genetic variation revealed that the average number of substitutions in isolate 1p49 was significantly higher compared to the other isolates (*p* value = 2.6e^−15^) (Figure 4). In terms of amino acid substitutions, DK-9895174 and 1p49 displayed the highest variability. This was even more evident when comparing numbers of substitutions after quality filtration only (Table 4).

## Discussion

In general, resequencing projects rarely exceeds a read depth of 100×, which barely exceeds the depth needed in order to correct for sequencing errors. In this study, we established a protocol which provided an average coverage of the four analysed VHSV genomes of between 0.5 and 1.9 million reads, while low coverage areas were observed only in the initial part of the leader region, as well as in the trailer region.

The results clearly demonstrated that the four isolates of VHSV were composed of a genetically diverse virus population, the first requirement for the formation of a quasispecies. Intra-host genetic variation has been demonstrated previously for other rhabdoviruses such as rabies virus [22] but to our knowledge, this study is the first to describe the intra-host genetic diversity in a novirhabdovirus using ultra deep sequencing.

**Figure 5:**
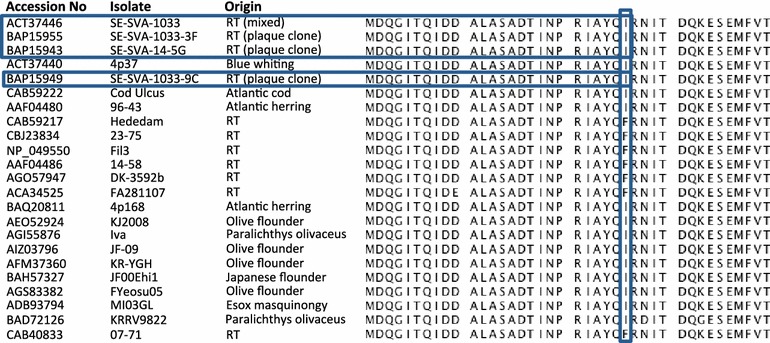
**Alignment of all L protein sequences available in GenBank.** Amino acid position 1012 is highlighted by the vertical box.

Statistical comparison of local substitution rates across genes revealed that the G and L genes comprised the majority of the genetic variation and showed significantly different substitution rates compared to the N, P, M and NV genes. These findings are likely related to the essential functions of these proteins: The G protein is exposed on the surface of the virus particle and involved in receptor recognition as well as cell entry. At the same time, G is the target of neutralizing antibodies. Selection pressure on G thus includes both the need for ability to adapt to variation in host cell surface molecules as well as ability to escape from the host adaptive immune system [28]. The L gene is responsible for genome replication (transcription and translation), hence the observed higher genetic variation may indicate the need to be able to adapt to replication conditions in different cell types or host species. Recently, a single amino acid substitution (I1012F) in the L protein was shown to be associated with a change in the in vitro virulence of an isolate of VHSV obtained from marine fish. Following the substitution, this isolate was able to replicate in primary cultures of rainbow trout gill epithelial cells. While the parental isolate was avirulent to rainbow trout, virulence of the recombinant variant in vivo was not analysed [29]. The present study included two isolates originating from wild Atlantic herring (1p49 and 4p168) that are known to be avirulent for rainbow trout [8, 30]. Sequences of these isolates were mapped against those of an isolate of VHSV originating from the marine environment. However, this reference isolate (FA281107) originated from sea-reared rainbow trout and represents the first VHSV isolate of genotype III pathogenic to rainbow trout [13]. In both herring isolates, isoleucine (I) was detected at amino acid position 1012 in the L protein within the list of quality-filtered variants at a frequency higher than 99%, while the marine reference isolate known to be pathogenic to rainbow trout showed a phenylalanine (F) at this position. To further investigate this aspect, we aligned the 23 VHSV L protein sequences currently available at NCBI, and found a general correlation with the presence of the I1012F substitution when the host is rainbow trout (Figure 5). There were, however, four exceptions which included the virulent SVA-1033 isolate and two plaque clones of the same isolate and a plaque clone of another mixed isolate (SVA-14). Based on typing using monoclonal antibodies, the Swedish isolates SVA-14 and SVA-1033 contains a mixed virus population (N. Lorenzen unpublished data) so the available Sanger consensus sequences might therefore be misleading, and the reason why plaque-cloning attempts have been performed. Our findings show the same trend as the in vitro findings reported by Kim et al. [29], although it remains to be proven e.g. by reverse genetics that the single substitution I1012F facilitates an increase in the in vivo virulence of a marine strain of VHSV for rainbow trout. Statistical comparison of local substitution rates across isolates reveal that the 1p49 isolate indeed behaved differently compared to the other three isolates. The high number of nucleotide substitutions might indicate a low and unstable state within the fitness landscape. While it cannot be excluded that the higher stability of 4p168 isolate could be due to passage of the latter in one cell line only (BF2) compared to two for 1p49 (BF2 and EPC), the difference might also reflect different host/environmental conditions at the geographical sites of virus isolation. Both marine isolates were obtained from tissue pools of herring. However, while isolate 4p168 was from Skagerrak, a region with stable high salinity, limited fish species diversity and low prevalence of VHSV occurring in only a few species, isolate 1p49 was from the Baltic Sea, characterized by high fluctuations in salinity and with rather high prevalence of VHSV in a range of different fish species [31]. In terms of the two freshwater VHSV isolates, both derived from serious disease outbreaks in freshwater-farmed rainbow trout, the observed differences in variability might also be related to host conditions. Although isolate DK-3592B displayed slightly higher nucleotide variability following frequency filtering, this was not reflected at the amino acid level, where isolate DK-9895174 displayed the highest variability. When looking at the data after quality filtering but before frequency filtering, the higher variability of DK-9895174 became even more prominent (Table 4). While isolate DK-3592b was derived back in 1985, where almost all Danish VHSV isolates belonged to a single serotype (based on a plaque neutralization test), isolate DK-9895174 was isolated 14 years later, when serotyping data revealed a higher frequency of isolates not neutralized by reference antibodies raised against the original VHSV F1 isolate [32, 33]. To confirm whether our NGS data reflects viral quasispecies related adaptations to selective pressures set by the host availability or immune defence as discussed above will require more extensive analyses.

Errors may occur at any of the many steps involved in deep sequencing of the evolving pathogen population, including RNA extraction; reverse transcription; PCR amplification of target regions; library preparation and sequencing; read quality control and filtering and mapping. In a deep sequencing analysis of a mixture of HIV clones, the estimated PCR chimera rate was 1.9% [34], while the average error rates when using the Illumina platform have been estimated at between 0.31 and 1.66% [35]. Estimation of the actual accumulated error rate in the performed experimental setting is not possible at this point, a number of internal controls (e.g. sample replicates) is needed for this. Instead we filtered using conservative quality parameters as well as ignored variants at frequencies below 5%.

In the present study, 0.5–1.9 million × coverage was obtained. However, during the process of data analysis, we realized that a number of control samples are needed in order to be able to determine that mutations detected at extremely low frequencies (under 1%) represent true polymorphisms. Such controls could consist of plaque-cloned viral samples and control plasmids. These would provide the baseline for the error rate due to the methods used. Once determined, the established deep-sequencing methodology would prove very powerful for analysis of tissue samples from viral adaptation studies. With respect to the actual sample source, the use of an enrichment approach, either by hybridization [36, 37] or through enriching for virus-specific RNA by depleting host genomic DNA and rRNA, might be relevant to explore as well [38]. Nevertheless, since all virus isolates had been propagated in vitro, our results may not fully reflect how VHSV acts as a quasispecies in vivo. This will need further deep sequencing analyses of VHSV genomes directly derived from infected fish.

The overall aim of this study was to better understand the potential for evolution and host adaption of VHSV by applying a deep-sequencing approach. Based on the multiple and unique results obtained during this study, we find that the established deep sequencing and applied bioinformatics- and statistical analysis methods are valuable, and will probably be even more when used in future in vivo host adaption studies of VHSV.
